# To Assist Oncologists: An Efficient Machine Learning-Based Approach for Anti-Cancer Peptides Classification

**DOI:** 10.3390/s22114005

**Published:** 2022-05-25

**Authors:** Majed Alsanea, Abdulsalam S. Dukyil, Afnan, Bushra Riaz, Farhan Alebeisat, Muhammad Islam, Shabana Habib

**Affiliations:** 1 Computing Department, Arabeast College, Riyadh 13544, Saudi Arabia; malsanea@arabeast.edu.sa; 2 STC Academy, Riyadh 13315, Saudi Arabia; adukyil@stc.com.sa; 3 Digital Image Processing Laboratory, Department of Computer Science, Islamia College University Peshawar, Peshawar 25000, Pakistan; afnanstd@icp.edu.pk; 4 Department of Biomedical Science, Ajou University School of Medicine, Suwon 16499, Korea; bushra0869@ajou.ac.kr; 5 Information Technology Department, ICT College, Tafila Technical University, Tafila 66110, Jordan; fobisat@ttu.edu.jo; 6 Department of Electrical Engineering, College of Engineering and Information Technology, Onaizah Colleges, Unaizah 56219, Saudi Arabia; m.islam@oc.edu.sa; 7 Department of Information Technology, College of Computer, Qassim University, Buraydah 52571, Saudi Arabia

**Keywords:** anticancer peptides, artificial intelligence, biomedicine, statistical approach, machine learning

## Abstract

In the modern technological era, Anti-cancer peptides (ACPs) have been considered a promising cancer treatment. It’s critical to find new ACPs to ensure a better knowledge of their functioning processes and vaccine development. Thus, timely and efficient ACPs using a computational technique are highly needed because of the enormous peptide sequences generated in the post-genomic era. Recently, numerous adaptive statistical algorithms have been developed for separating ACPs and NACPs. Despite great advancements, existing approaches still have insufficient feature descriptors and learning methods, limiting predictive performance. To address this, a trustworthy framework is developed for the precise identification of ACPs. Particularly, the presented approach incorporates four hypothetical feature encoding mechanisms namely: amino acid, dipeptide, tripeptide, and an improved version of pseudo amino acid composition are applied to indicate the motif of the target class. Moreover, principal component analysis (PCA) is employed for feature pruning, while selecting optimal, deep, and highly variated features. Due to the diverse nature of learning, experiments are performed over numerous algorithms to select the optimum operating method. After investigating the empirical outcomes, the support vector machine with hybrid feature space shows better performance. The proposed framework achieved an accuracy of 97.09% and 98.25% over the benchmark and independent datasets, respectively. The comparative analysis demonstrates that our proposed model outperforms as compared to the existing methods and is beneficial in drug development, and oncology.

## 1. Introduction

Oncology is a medical specialization that focuses on the diagnosis and treatment of persons having cancer. Cancer is the most debilitating illness and the leading cause of mortality in both economically developed and undeveloped countries. This deadly illness claims the lives of over eight million individuals each year [1]. According to forecasts, the number of cancer cases is expected to increase to 16 million by 2020 [2,3]. Cancer treatment using traditional procedures, such as chemotherapy, radiation therapy, hormone therapy, and targeted therapy, has been judged to be ineffective owing to the high expense and detrimental effects on normal cells [4,5].

**Figure 1:**
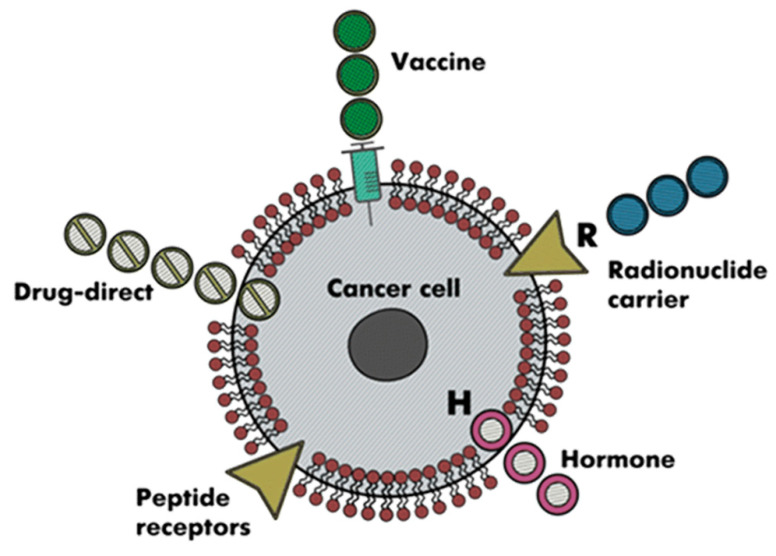
Various possible treatment options for cancer using peptides sequence.

ACPs have been regarded as the most effective cancer treatment over the past several decades due to their inability to interfere with normal body physiological activities. Different potential treatment possibilities for cancer that target peptides as shown in Figure 1. They have been utilized in pre-clinical studies for a variety of objectives, including diabetes, cardiovascular illness, and several types of malignancies [6,7]. ACPs offer extraordinary and distinct advantages, such as being more efficient and less dangerous than synthetic medications [4]. A peptide’s sequence is comprised of less than 50 amino acid residues. ACPs deal extraordinary and distinct advantages, such as being more efficient and less dangerous than synthetic medications. ACPs are easily able to treat cancerous cells because of their amphiphilic nature however the specific affected cells are removed by engaging them with anionic cell membrane components of a cancer cell [8].

Early-stage cancers have a greater chance of survival and are less likely to cause morbidity [9]. In the healthcare system, the failure to diagnose cancer at an early stage can pose a significant problem in treating patients. Cancer is not accurately detected due to insufficient noninvasive and accurate markers [10]. Peptide-based biomarkers have contributed to the earlier detection of cancer because of advancements made in genomics and proteomics [11]. Once cancer has been diagnosed, treatment is the next step. In the current medical system, conventional cancer treatments include chemotherapy, radiation therapy, hormonal therapy, and surgery. Traditional treatments are limited by unfavorable side effects and high expenses [12]. The possibility of cancer occurring again after successful treatment, means we need a better and more effective treatment [13]. Currently, peptide-based therapies have emerged as a novel treatment strategy for cancer [4]. These features include high specificity, good efficacy, easy synthesis, low toxicity, chemical modification ease [14,15], and less immunogenicity in comparison with recombinant antibodies. Recent research has indicated that therapeutic peptides can be used both as a diagnostic tool and as a potential treatment for many diseases [16,17]. The last decade has reported many natural peptides, which possess diverse biological activities (antifungal, antiviral, antibacterial, anticancer, tumor-homing) [18].

Several recent articles [19,20,21,22] have shown that applying the principles of Chou’s 5-step procedures while building a new sequence-analyzing tool or statistical predictor: (a) The first phase in constructing a predictor is to identify or design a valid benchmark dataset; (b) The second phase related to organizing the data in a way where the internal connection for target peptide is detected that may be correctly reflected; (c) The next phase is to choose the optimum operating model; (d) The model is then assessed over test data using various evaluation metrics in the fourth phase; (e) and eventually, a user-friendly and publicly available web-server for the predictor is launched. We deeply investigated the literature study and found two main problems:

- Prior peptide classification models were developed using a single feature descriptors method without any modification that captured meaningless information against each peptide sequence.
- To improve the accuracy of peptide classification, most of the studies followed a fusion strategy to collect a diverse and massive number of features, resulting in homogeneous patterns and high dimension descriptors that affect the model performance.

To overcome these problems, we built an effective and computationally intelligent framework for ACPs prediction. To express peptide sequences, three unique protein sample formulation approaches are used: amino acid composition (AAC), dipeptide composition (DPC), tripeptide composition (TPC), and an improved version of IPseAAC. In addition, PCA is used to find strong discriminatory features from extracted feature spaces. Finally, three numerous classification learners, such as support vector machine (SVM), random forest (RF), and naïve Bayes (NB), are ensembled as operational algorithms to test the proposed model’s predicted outputs. Our four-fold contributions are the following bullets:

- Due to the lack of an effective vaccine, the increase in drug-resistant, and the fatal nature of cancer, we present a novel intelligent framework for efficiently distinguishing anticancer peptides from unstructured peptides sequences. The proposed paradigm is beneficial to the development of vaccines against cancer peptides.
- The variety and numbers of peptides in databanks are rapidly increasing due to the advancement of sequence technology. We deeply investigated the literature and concluded that most of the researchers use numerous flavors of encoding techniques, which exhibit poor performance when extracting contextual information from peptide sequences, resulting in non-representative algorithms. In this paper, a novel approach is presented that engages a diverse collection of features to obtain using statistical methods. The proposed model extracts contextual features to accurately categorize the nature of peptides.
- The PseAAC method demonstrates an incredible performance in various protein sequence classification that comprises three physicochemical properties including hydrophilicity, hydrophobicity, and charge of basic amino acids however, sometimes it gives poor results when the sequence of peptides is short in length. To improve the prediction strength, we added some new physicochemical properties including flexibility, irreplaceability, solvent accessible surface area, polarity, polarizability, and rigidity.
- We performed numerous possible combinations of features against an ensemble classifier to evaluate the strength of individual components. The proposed model shows convincing results and provides new state-of-the-art (SOTA) accuracy over testing peptide sequences.

The rest of the article is arranged as follows: Section 2 provides a brief description of the existing studies, while Section 3 covers materials and procedures. Similarly, in Section 4 comprehensive experimental results are briefly discussed. Finally, Section 5 concludes the study with a future research plan.

## 2. Related Work

In this section, we demonstrate the use of well-known techniques for the classification of ACPs based on traditional machine learning (ML) methods. As demonstrated in Table 1, all works discriminate peptides into two categories: positives (ACPs) and negatives (NACPs).

Manually experimentation strategy to identify new ACPs is time-consuming and costly. As ACPs play a crucial role, therefore academics and pharmaceutical companies have turned to automation as an alternative method for identifying ACPs. In this regard, researchers have used a variety of automated intelligence algorithms to predict ACPs. In an anticancer study [23], Chen et al. presented the “iACP” framework for peptide identification. They used an improved G-Gap DPC in conjunction with peptide sequence formulation. Similarly, Manavalan et al. developed a novel model for predicting ACPs [24]. The composite feature set, on the other hand, is made up of optimal information that includes physicochemical properties, DPC, ionic, and so on. K-fold cross-validation is used to train and test the proposed system. Furthermore, Tyagi et al. created silico algorithms to discriminate ACPs from uncharacterized sequences [30]. Four separate datasets are used to evaluate the peptides classification model. On the other hand, two statistical methods including split AAC and binary profile are applied for peptides encoding. Although, Li et al. introduced a mechanism for feature integration to discriminate of ACPs [31]. To extract robust features, a compact form of AAC, properties of individual amino acids, and traditional AAC are used. Using SVM, the predictor model increased its performance in terms of accuracy. Akbar et al. created a new model named “iACP-GAEnsC” to identify ACPs [32]. They followed a hybrid encoding strategy to extract high representative features from target peptides. An evolving genetic algorithm is used to assess the performance consequences of the created technique. Kabir et al. created “TargetACP,” revolutionary adaptive genetic algorithms and sequential facts [33]. Furthermore, the synthetic minority oversampling technique is a practice that efficiently distributes samples between minority and majority classes into equal sizes. The proposed system was tested using two different benchmark datasets and yielded better performance results. Moreover, Kumar et al. introduced a web server namely “ACPP” that precisely identifies the positive peptides from negative samples [34]. Their system revealed many settings that allow the operator to construct and identify ACPs properly. It can also give information about the lethal function of each target peptide. Likewise, Hajisharifi et al. predicted ACPs using pseudo amino acid composition (PseAAC) and a unique kernel with local alignment [35]. In a subsequent study, Xu et al. used the g-gap DPC approach to peptide encoding [25]. To reduce unnecessary and homogenous features they utilized maximum relevance-maximum distance. To further boost the performance Boopathi et al. presented two variant feature selection techniques that choose optimal yet informative descriptors from features space generated via seven peptide encoding methods [26]. Most of the ML models show inadequate performance due to high dimensional descriptors. To address such an issue Li et al. proposed a model based on various feature extraction techniques and obtain incredible performance when forwarding a 19-dimensional vector [27]. Due to the diverse nature of genomic sequences, accurate classification of target peptides has become a challenging job, therefore Akbar et al. fused three peptide encoding methods, and later k-space amino acid pairs were applied to extract more correlated features [28]. In an another study, Agrawal et al. analyzed the performance of the ETree classifier with AAC and DPC, and based on the best model they designed a webserver that is compatible with edge devices [29].

Due to the importance of medicine and the growing trend in the application of ML techniques, we briefly presented a literature review that discusses how these techniques are used for cancer prediction and prognosis. Researchers in these studies consider prognostic and predictive factors independent of treatment, or they integrate these factors to guide treatment for cancer patients. Furthermore, we present the different types of ML methods that are used, the types of data they incorporate, as well as the pros and cons of each technique. By utilizing ML and artificial intelligence, precision medicine-based treatments can become more targeted. To make medical predictions, researchers should develop their understanding of cause and reflect on relationships between factors such as how a cancer patient responds to drug treatments.

## 3. Materials and Methods

**Figure 2:**
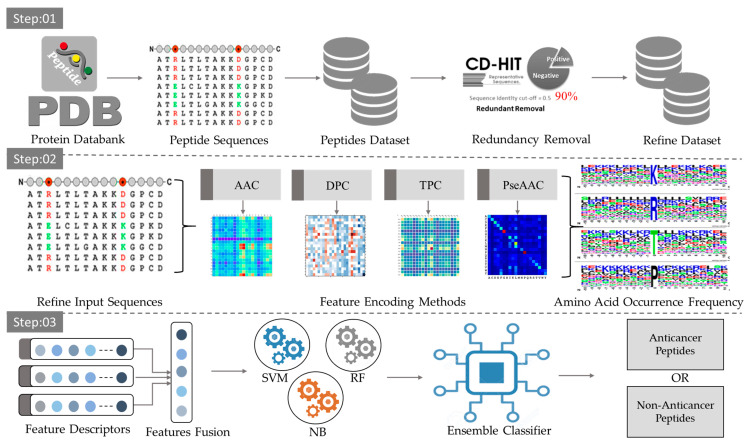
The proposed framework for the classification of ACPs and NCPs.

In this section, all the components used in the proposed work are briefly described. The overall mechanism is presented in Figure 2.

### 3.1. Dataset

For statistical predictors, selecting or creating a trustworthy dataset is crucial since it has a significant impact on classification measures. Maintaining the significance of the dataset in mind, two well-known datasets, namely the benchmark and the main datasets are used in this study for the experimental purpose [30]. Although, these datasets are divided into two categories: ACPs and NACPs, where the ACPs data is taken from the anuran defense peptides database [36] and the antimicrobial database and peptides [37]. Conversely, NACPs biological sequences are obtained using an arbitrary selection of peptides from the Swiss-Prot proteins databank [38]. The main dataset has a total of 2475 sequences, containing 225 ACPs and 2250 NACPs. [39]. The benchmark dataset, on the other hand, was collected from [23] which contains 138 ACPs and 206 NCPs [30]. In this study, the main and benchmark datasets are represented by (PSD_1_) and (PSD_2_). The set of ACPs is represented as S_1_+ and a set of NCPs is represented by S_2_-. The union and intersection of the $S^{+}$ and $S^{-}$ express benchmark dataset in Equations (1) and (2).

(1) $$D_{1}=S_{1}^{+}{\;U\;S}_{2}^{-}\;$$

The intersection of the $S^{+}$ and $S^{-}\;$ of the data set should be empty.

(2) $$D_{1}=S_{1}^{+}\;\cap {\;S}_{2}^{-}\;$$

The format of the AntiCP sample in the dataset is given below:

>ACP_1 GLWSKIKEVGKEAAKAAAKAAGKAALGAVSEAV

### 3.2. Preprocessing

To get the best outcome and accuracy, preprocessing removes duplication and noisy data from the data using several tools like Jalview and cluster database at high identity with tolerance (CD-HIT). In this work, a CD-HIT tool is used to reduce repeated peptides and similarity bias. According to the general homology bias, peptides with more than 90% identical sequences are discarded.

### 3.3. Peptide Encoding Methods

The most challenging task in the post-genomic era is to determine how to generate a biological sequence with a discrete model that preserves important sequence-order information or a crucial motif feature. As shown in a comprehensive study, ML techniques (such as the ‘Optimization’ method [40], the ‘Covariance Discriminant’ algorithm [19], the ‘K-Nearest Neighbor (KNN)’ [41] algorithm, and the SVM algorithm [42] can only employ vectors. The loss of sequence-motif information is the key concern noted in the discrete model. Chou proposed PseAAC to maintain sequence-motif information in protein [20]. In the domain of computational proteomics, the PseAAC method has been widely used for feature extraction purposes [22]. Considering the PseAAC technique, four strong open-access software packages were created: ‘PseAAC’ [43], ‘PseAAC-Builder’ [44], ‘propy’ [45], and ‘PseAAC-General’ [46]. The initial three are mainly used for creating different characteristics of Chou’s unique PseAAC [47], whereas the final is for Chou’s standard PseAAC [48]. Such a method not only extracts specific and fixed-length features but can capture some higher-level features including ‘functional domain’ mode, ‘gene ontology’ mode, and ‘PSSM’ mode. Considering the encouraging success of PseAAC in the field of proteins, it was expanded to DNA and RNA using the PseKNC idea (Pseudo K-tuple Nucleotide Composition) [49]. ‘Pse-in-One’ [50], a strong web server, and its upgraded version, ’Pse-in-One2.0,’ [38] were recently released. The statistical details of the datasets used in this research is mentioned in Table 2, where the total samples are split into standard 70% and 30% for training and testing, respectively.

The input data is transformed into numerical descriptors in the feature extraction process, which describe different information about the peptides [51]. To reliably identify protein sequences, many algorithms have been presented in the early studies to capture high discriminative features [52]. In this study, four diverse peptide encoding techniques, namely: AAC, DCP, TPC, and IPseAAC are capable to gather salient, robust, and meaningful information from input biological sequences.

#### 3.3.1. Amino Acid Composition (AAC)

The peptide is made up of a 20-amino-acid sequence. There are two sorts of models that may be used to describe a peptide sequence: sequential and discrete models. However, we only focused on discrete models in our research where AAC is the most basic and often used for biological sequence classification. The AAC of a peptide sequence is made up of 20 distinct integers that indicate the standardized frequency of occurrence of 20 basic amino acids in peptides. When AAC is applied to peptides we can get a 20D vector as shown in Equations (3) and (4). Let’s assume P is a peptide sequence containing N amino acids.

(3) $$P=\left[{Ҏ}_{1},\;{Ҏ}_{2},\;{Ҏ}_{3},\;{Ҏ}_{4},\ldots .,{Ҏ}_{N}\right],\;\mathrm{where}\;{Ҏ}_{i}\;\epsilon \;R=[A,\;C,\;D\ldots .\;Y]$$

(4) $$P_{i}=\frac{n_{j}}{L},\;j\;\epsilon \;\left[1,2,\;3,\;4,\ldots ,20\right],$$

where n_j_ is the number of times a certain amino acid appears in a protein sequence of length L so, finally AAC can be further formulated using Equation (5):

(5) $$P=\left[{Ҏ}_{1},\;{Ҏ}_{2},\;{Ҏ}_{3},\;{Ҏ}_{4},\ldots .,{Ҏ}_{N}\right]T,$$

where, P_i_ ϵ R = [A, C, D…. Y] illustrates the appearance frequencies of 20 native amino acids while T is the transpose function that arranges data row/column-wise. The lack of sequence-length effects, which ignores exact hidden information in protein sequences, is the fundamental shortcoming of AAC-based features. To overcome this problem Chou introduced the idea of PseAAC.

#### 3.3.2. Dipeptide Composition (DPC)

For the encoding of cancer peptide sequences, DPC is a discrete technique that mainly considers neighbor amino acid features to train the ML algorithms. It represents the occurrence number of adjoining amino acids and finally generates a 400D vector against each peptide. It provides details about protein sequences on a massive level. The fundamental benefit of DPC over traditional AAC is that it focuses on all the features of peptides, whereas AAC simply considers the single frequency of amino acids in peptides sequences. The feature descriptor for DPC can be computed via Equation (6):

(6) $$\mathrm{DPC}\left(i\right)=\frac{1}{{\mathrm{Total}\;}_{\mathrm{Feature}}}\left(\mathrm{DP}\left(i\right)\right),$$

where DPC(i) represents the overall frequency of each couple motif, DP(i) represents one single occurrence from 400 patterns, and total numerical values signify the whole collection of features.

#### 3.3.3. Tripeptide Composition (TPC)

The ability to extract a collection of meaningful parameters is one of the most crucial components of pattern recognition. In biology, tripeptides are vital peptide encoding mechanisms that capture silent and discriminative features. A good and minimum biological recognition signal consists of three consecutive amino acids. This might serve as a model for identifying peptides and tiny organic molecule mimics that can be used as biological function modulators. The early study has shown that the tripeptide can be used to anticipate probable oligopeptide structures and to create new peptides. As a result, tripeptide compositions were used to represent membrane protein samples in this study. We computed the probability of each tripeptide appearing in the peptide sequence using Equations (7) and (8) and scanning one sequence using a sliding window of three residues in one step.

(7) $$f_{i}=\frac{n_{i}}{\sum_{i=1}^{8000}n_{i}}=\frac{n_{i}}{\left(L-2\right)},$$

where the total number of the ith tripeptide and length of the sequence are represented by n_i_ and L respectively. The peptides may be stated as follows using an 8000-D feature vector:

(8) $$F_{8000}=\left[f_{1},{\;f}_{2},{\;f}_{3},\ldots f_{8000}\right]T$$

where transpose of feature vector and the frequency of each pattern in *i*th tripeptide is demonstrated by T and f_1_, respectively.

#### 3.3.4. Improved Pseudo Amino Acid Composition (IPseAAC)

Early peptides studies reveal that the amino acid sequences that make up proteins have been linked to the structure and function of proteins in studies. To assist the rapid growth of protein subcellular location prediction, researchers have presented a variety of feature extraction approaches and created associated web servers and software. Chou’s PseAAC, broadly employed in protein-protein interaction prediction and subcellular position prediction, takes into consideration the order information of proteins and the physicochemical characteristics of amino acids. The amino acid sequence of a protein is represented by (20 + *λ*) a dimensional vector in PseAAC. The first 20 dimensions indicate the frequency of occurrence in the sequence of the traditional 20 types of amino acids, whereas the other dimension reflects sequence-related parameters that depict differing amounts of amino acid sequence information.

The peptide sequence is encoded using an IPseAAC in this research. In the realm of genomics, AAC has been used to identify a variety of proteins and peptides, however, the identification process might be improved by adding some physiochemical features to AAC. The following equations show the IPseAAC feature extraction process:

(9) $$P={\left[a_{1},a_{2},\ldots a_{20},a_{20+1},a_{20+2},\ldots .a_{20+\lambda }\right]}^{t}\;\left(\;\lambda =1,\;2,\ldots \ldots .21\right)$$

In Equation (9), *a*_1_, *a*_2_, *a*_3_………. *a*_20_ indicates the frequency of twenty amino acids while the rest represents the correlation factors of amino acids including, hydrophobicity, hydrophilicity, charge properties, flexibility, irreplaceability, solvent accessible surface area, polarity, polarizability, and rigidity of amino acid. These characteristics of amino acids play a crucial role in peptide categorization. Physiochemical features are added via a variety of approaches. Numerous parameters are used to predict the peptide sequence. In this work, we experimented with several values of *λ* but found that *λ* = 1 yielded the best results. Some basic formulas for computing the correlation among physicochemical properties are given in Equation (10):

(10) $$\left\{\begin{array}{c} \lambda 1=\frac{1}{length-1}\overset{length-1}{\underset{k=1}{\sum }}I_{k},k+1\\ \lambda 2=\frac{1}{length-2}\overset{length-2}{\underset{k=1}{\sum }}I_{k},k+1\;\\ \lambda 3=\frac{1}{length-3}\overset{length-3}{\underset{k=1}{\sum }}I_{k},k+1\\ \lambda 4=\frac{1}{length-4}\overset{length-4}{\underset{k=1}{\sum }}I_{k},k+1\\ \lambda 5=\frac{1}{length-5}\overset{length-5}{\underset{k=1}{\sum }}I_{k},k+1\\ \ldots \ldots \ldots \ldots \ldots \ldots \ldots \ldots \ldots \ldots \ldots \ldots \ldots \ldots \ldots \ldots \ldots .\\ \lambda n=\frac{1}{length-n}\overset{length-n}{\underset{k=1}{\sum }}I_{k},k+1 \end{array}\right.$$

where the length encounters the total amino acids in the peptide sequence with a diverse factor of *λ* at variant ranks, respectively.

### 3.4. Optimal Feature Selection Technique

Nowadays, artificial intelligence-related research shows tremendous performance in numerous domains such as protein analysis [53], surveillance data [54,55], and power prediction [56,57,58,59,60,61]. The generated feature vector is extremely significant in ML and is efficiently utilized to forecast biological datasets. However, high-dimensional feature spaces might lead to erroneous and poor classification outcomes. Furthermore, training and testing a proposed approach requires a large amount of computing time and memory. Various feature selection strategies have been used to minimize the feature space to solve these challenges. Feature selection is a method of reducing redundant and unnecessary features to enhance prediction accuracy. The feature selection in this model is accomplished using PCA. The number of associated characteristics is reduced via PCA to a limited number of uncorrelated attributes. Principal components are the random variables that have been calculated. The primary benefits of PCA are that it reduces the dimensionality of a feature vector while minimizing correlation and meaningful feature loss. PCA’s global euclidean structure makes it more susceptible to outliers.

Assume a feature vector ‘*X*’with dimensions of P*Q, where ‘*P’* denotes the number of extracted features, ‘*Q’* denotes the number of peptide sequences, and *‘K’* is the feature vector’s needed dimension. The value of ‘*K*’ must be less than the value of ‘*Q’*. PCA employs the following procedures to reduce dimensionality using Equations (11) to (15):

(a) The average value of each attribute can be calculated as:
  (11) $${\bar{X}}_{j}=\frac{1}{P}\sum_{i=1}^{P}X_{i}$$
(b) The gap between the average values of X and X_i_:
  (12) $$\delta_{i}={\;X}_{i}-\bar{X}$$
(c) The covariance matrix can be calculated as:
  (13) $$C_{m}=\left(X_{i}-\bar{X}\right){\left(X_{i}-\bar{X}\right)}^{T}BB^{T}$$
  where B = {$\delta_{1},{\;\delta }_{2,\ldots \ldots ..,}\delta_{P}$}in(Q*P)
(d) The eigenvalue of *C_m_* is computed as:
  (14) $$\partial_{1}>\partial_{2}>,\ldots \ldots ,\partial_{N}$$
  where the largest eigenvalues ‘*∂_1_*’should be less than the highest of second ‘*∂*_2_’ and so on.
(e) Evaluate the eigenvector as:
  (15) $$C_{m}:V_{1},{\;V}_{2},\ldots \ldots ..,V_{N}$$

### 3.5. Classification Algorithms

#### 3.5.1. Support Vector Machine (SVM)

SVM is based on statistical learning theory, and it was initially used for binary classification problems instead of multiclass classification. In the case of a binary, SVM turns input into a high-dimensional feature vector to find the best hyperplane. To quantify classification power, SVM employs a variety of kernel functions, including linear, polynomial, RBF, and sigmoid. To investigate the benchmark datasets, the RBF kernel function is utilized in this work, using two parameters: ‘C’ and ‘Y’ that can be calculated via grid search and optimization procedures. Mathematically the RBF kernel function is defined in Equation (16):

(16) $$K(x_{i},{\;x}_{j}=\exp{\left(-Ɣ\left|x_{i}-x_{j}\right|\right)}^{2}$$

#### 3.5.2. K-Nearest Neighbor (KNN)

In the fields of ML and pattern recognition, KNN is an instance-based categorization algorithm that has been successfully employed. KNN is a non-parametric technique that does not use any previous knowledge about the training data to frame any complete model. KNN classifies a data sample into the class that appears to be the most persistent among its nearest neighbor samples. It measures the distance between instances of a feature space using the Euclidian distance. The distance between two points can be calculated using the Equation (17):

(17) $$D_{Euclidean}=D\left(X,Y\right)=\overset{n}{\underset{i=1}{\sum }}{\sqrt{\left(x_{i}+x_{j}\right)}}^{2}$$

where *X* and *Y* are two observations from the training and testing sets; *x_i_* and *x_j_* are two input variables in the same set.

#### 3.5.3. Random Forest (RF)

RF is a supervised learning algorithm capable of assessing both binary and multiclass issues by default. RF constructs numerous decision trees using a statistical Bootstrap approach based on a random selection of data samples from training data. As a result, a “forest” with a great number of trees is produced. To discover the optimal split at each node of the tree, various numbers of predictors are utilized. RF’s ability to remove biases and minimize correlation among unpruned trees was aided by his random selection nature. Finally, using the majority voting approach to combine the predictions of each unique assumption, an optimal output is generated. There are 100 trees and 200 iterations in this work.

#### 3.5.4. Ensemble Classifier Mechanism

The training and testing procedure on extracted feature vectors is one of the most significant parts of data mining, ML, and bioinformatics. Due to superior predictive accuracy, an ensemble classifier has a more favorable reception than an individual classifier. For several computational models, ensemble classification has been suggested. The ensemble classifier reduces the discrepancy caused by irregularity in an individual training set, making it superior to an individual classifier. We presented a three-classifier combination in this study: SVM, RF, and NB as given in Equation (18).

(18) $$E_{ensemble}=SVM⊕RF\;⊕NB$$

The ensemble classifier, which uses a voting mechanism to merge three independent classifiers, is shown in Equation (18). The margin operation was marked by (⊕). For the combination of three classifiers, the ensemble classifier *E_ensemble’s_* complete procedure is as follows. Let’s look at a single classifier’s predicted performance for identifying ACP and NACP.

(19) $$(C_{1},C_{3},C_{3})\;E\;\left(\;A_{1},A_{2}\right)$$

In Equation (19), individual classifiers are represented as (*C*_1_, *C*_2_, *C*_3_), and (*A*_1_, *A*_2_) have specified ACP and NACP classes, respectively.

Finally, the ultimate result of the *E_nsemble_* utilizing the voting process is given in Equation (20).

(20) $$E_{ensemble}=Maxi\left(w\left(1\times 1\right),w\left(2\times 2\right),w(3\times 3\right)$$

where *E_nsemble_* is the ensemble method using the voting process, *Maxi* is the highest accomplishment, and *w*_1_, *w*_2_, and *w*_3_ are the best weights of the several classifiers. Finally, as a result classifier predicts the class has maximum votes.

### 3.6. Model Evaluation

In this section, the first system specification for the proposed system is discussed with the dataset division for training and testing. Secondly, the proposed model is evaluated via numerous evaluation metrics to compute the complementary power of the model.

#### 3.6.1. System Configuration and Data Setting

All the experiments were conducted using MATLAB (2020a) installed in GeForce GTX 2060 GPU having 64GB RAM. Before exercising the model, preprocessing is performed where entire peptide sequences of each dataset are passed via CD-HIT software to remove the high similarity score, and later the refined data is divided into 70% (training) and 30% (testing).

#### 3.6.2. Evaluation Metrics

Different factors are used to measure the success of an intelligent predictive algorithm in ML. The classification method’s true and false projected outcomes are kept in a confusion matrix. Typically, accuracy is used to assess the strength of hypothesis learners in various assessment approaches, however, accuracy alone is insufficient to assess a prediction model’s effectiveness. Moreover, a set of four metrics based on Chou’s symbols for examining protein signal peptides were proposed, and these metrics were later adopted by several publications. But the provided metrics are only applicable for single-label networks; multi-label systems (where data may belong to many classes at the same time) are more commonly seen in genetics, medicine, and biomedicine, which need completely other sets of metrics. The following performance metrics are used in this model to correctly assess ACPs and NACPs.

(21) $$Accuracy=1-\frac{ACP_{\_}^{+}+ACP_{\_}^{+}}{ACP_{\_}^{+}+ACP_{\_}^{+}}$$

(22) $$Sensitivity=1-\frac{ACP_{\_}^{+}}{ACP_{\_}^{+}}$$

(23) $$Specificity=1-\frac{ACP_{+}^{\_}}{ACP_{+}^{\_}}$$

(24) $$MCC=\frac{1-\frac{ACP_{\_}^{+}+ACP_{\_}^{+}}{ACP^{+}+ACP^{\_}}}{\sqrt{\left\{1+\frac{ACP_{+}^{\_}+ACP_{\_}^{+}}{ACP^{+}}\right\}\left\{1+\frac{ACP_{\_}^{+}+ACP_{+}^{\_}}{ACP^{\_}}\right\}}}$$

(25) $$F_{1}\_Score=2\times \frac{\left(Precision\times Recall\right)}{\left(Precision+Recall\right)}$$

In the above Equations, the ACP+ signifies anticancer peptides, whereas ACP- indicates non-anticancer peptides. ${\mathrm{ACP}}_{-}^{+}$ are anticancer peptides that have been mislabeled as another class label, while ${\mathrm{ACP}}_{+}^{-}$ are NACPs that have been misclassified as anticancer.

## 4. Experimental Results

In this section, a comprehensive ablation study is conducted over two famous datasets with various possible collections of techniques. Finally, the empirical results obtained through the proposed model are compared with the latest existing methods. A brief explanation about the individual section is provided in the sub-sections. 

### 4.1. Ablation Study over PSD1

To evaluate the individual component power of the feature extraction method we checked and compared a total of 13 models’ performance for sequence classification. The main purpose of comprehensive results is to find the discriminative, robust, and representative features so that training is smoothly performed. In the AAC encoding scheme total of 20 native amino acids frequencies are calculated but due to the dominance occurrence value problem, the performance is not convincing including 86.41, 88.24, 85.51, 0.71, and 81.08 in accuracy, sensitivity, specificity, Matthews correlation coefficient (MCC), and F_1_-score, respectively. DPC is our second computational-based method that boosted discriminative scores because it mainly maintains the correlation between two amino acids. The results obtained via DPC are accuracy 88.35, sensitivity 91.18, specificity 96.86, MCC 0.75, and F_1_-score 83.78. Next, the complementary power of TPC is evaluated where three consecutive amino acid patterns are scanned during the features extraction process, but the classification rate is less than the other two methods because the peptide sequence is too short therefore feature vectors mostly have zero values. Our last single feature extraction method is IPseAAC in which we added additional physicochemical properties that efficiently detected and discriminate the targeted peptide from complex biological sequences. The best score via IPseAAC is 88.35, 83.33, 91.80, 0.75, and 85.37 in accuracy, sensitivity, specificity, MCC, and F_1_-score, respectively. In this study, we also explored the feature fusion strategy where two individual peptide encoding approaches features are incorporated via a concatenation mechanism. An ensemble classifier is used throughout this study because it has strong discriminative power rather than an individual ML algorithm. Mostly in the literature studies, researchers investigated the single feature extraction method which works better for a simple and short length of peptides but fails in the case of huge or complex peptides. Therefore, a hybrid mechanism is explored having a diverse collection of feature extraction methods generated incredible results as compared to others. The main objective of integrating different method features is to examine its capability for lengthy and complex sequences. Among various fusion methods, the best performance is achieved when using DPC + IPseAAC including 93.20, 88.37, 96.67, 0.86, and 91.57 in accuracy, sensitivity, specificity, MCC, and F_1_-score. During this research, we discovered that feature fusion is the best approach to enhance the classification score but on the other side sometimes redundant features degrade the model performance.

**Figure 3:**
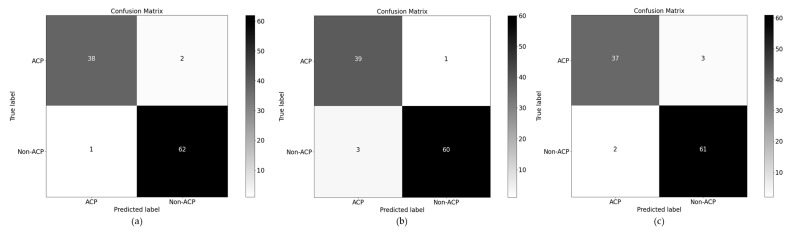
Performance evaluation of the proposed model over testing data of benchmark. Addition of physicochemical properties using the concept (**a**) (λ = 1); (**b**) (λ = 1); and (**c**) (λ = 1).

To address such an issue, we applied PCA that intelligently optimal features and ignore those attributes which have a low contribution rate in classification. In the proposed model four feature extraction methods features are fused and then employed PCA which gives outstanding performance along with different *λ* values as shown in Table 3. The confusion matrix for testing data is shown in Figure 3. 

### 4.2. Ablation Study over PSD2

**Figure 4:**
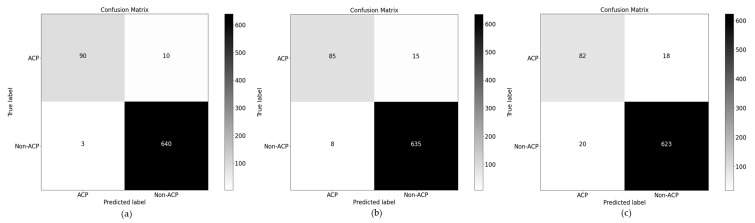
Performance evaluation of the proposed model over testing data of independent. Addition of physicochemical properties using the concept of (**a**) (λ = 1); (**b**) (λ = 1); and (**c**) (λ = 1).

We also conducted the ablation study over the second dataset to verify that either variant or a large number of sequences affect the model performance or not using the same feature extraction strategy. Through AAC we obtained 88.96, 58.82, 93.76, 0.53, and 59.41 in accuracy, sensitivity, specificity, MCC, and F_1_-score, respectively. On DPC we achieved good results. i.e., 91.25 accuracy, 68.42 sensitivity, 94.60 specificity, 0.61 MCC, and 66.67 F_1_-score, respectively. Like the first dataset, the performance of TPC here is also not too much better due to low pattern frequency and null values in the feature vector. The basic PseAAC contains three physicochemical properties while in this study we improved by adding additional attributes of each amino acid. So, through the improved version of IPseAAC we gained 90.98, 66.67, 94.72, 0.61, and 66.33 in accuracy, sensitivity, specificity, MCC, and F_1_-score, respectively. Among a hybrid collection of features method, DPC+TPC is also a tremendous classification score in terms of accuracy, sensitivity, specificity, MCC, and F_1_- score, which is 95.29, 84.21, 96.91, 0.79, 82.05 individually. At the same time, the combination of AAC+DPC our ensemble classifier attained 93.00, 75.53, 95.53, 0.69, and 73.20 in accuracy, sensitivity, specificity, MCC, and F_1_-score, respectively. Similarly, another hybrid model ACC+TPC also shows better results as compared to the previous one. In conjunction with ACC+IPseAAC, the classification rate is low because here homogenous features are rising on a large basis. Despite these, we proposed three different models with a varied number of λ which directly affect the overall discriminative system during the learning mechanism. The proposed model is investigated based on the (*λ* = 1, 2, and 3) wherein Table 4 results are demonstrating that on *λ* = 1 our ensemble classifier shows tremendous accuracy rather than others because it contains exhaustive numbers of physicochemical features. The confusion matrix for testing data is shown in Figure 4. 

### 4.3. Results Assessment with SOTA Methods over PSD1

In a comparative analysis, researchers used various feature extraction mechanisms and ML classifiers. For instance, Hajisharifi et al. [62] proposed an online tool for the classification of ACPs and NCPs where radial basis and Naïve Bayes functions are investigated in SVM. Their computational-based techniques show better performance on the limited number of peptides because they did not sufficiently extract features. Later Hajisharifi et al. [35] further enhanced prediction performance by using local alignment kernel and Chou’s pseudo amino acid with SVM. In their feature extraction, they applied three physicochemical properties where experimental results demonstrate that the model largely diverts to negative class rather than positive samples. Next, Chen et al. [23] introduced a novel biological sequence tool where g-gap dipeptide composition is optimized for discrimination of ACPs. To boost the prediction accuracy several researchers, explore the composite peptide encoding technique. For instance, Li and Wang [31] developed a model by integrating three feature extraction methods namely average chemical shifts, ACC, and reduced ACC with SVM. Their model captures redundant features; therefore, they obtained a high score on the jackknife test which is major flows. Despite these Akbar et al. [32], practice ensembled classifiers with hybrid feature space without investigating their performance and optimal peptide information. Similarly, Xu et al. [25] also proposed a hybrid model where g-gap dipeptide composition and maximum relevance maximum distance. Next, Li et al. [27] developed a lightweight model by considering low feature dimensional to address the time-consuming process. As we know peptide sequences mostly contain less than 50 amino acids, so classification is based on the ML method we need to generate more, and optimal features for precise prediction. Therefore, we deeply investigate the performance of each component of feature extraction and later evaluate the strength of the hybrid model with an ensemble classifier algorithm with an additional optimal selection technique. In this study, three different models’ performance is validated using the concept of diverse values of *λ.* The empirical results demonstrate that the proposed model with (*λ* = 1) obtain incredible classification accuracy as compared to other SOTA approaches as shown in Table 5.

### 4.4. Results Assessment with SOTA Methods over PSD2

For a fair comparison, it is necessary to match the results with SOTA techniques that used a similar dataset. Therefore, we deeply investigated the literature and found a total of four articles that evaluate their model on the same dataset. The first attempt is Tyagi et al. [30] proposed silico model based on binary profiles which obtain 92.65%, 74.67%, 94.44%, and 0.61 in accuracy, sensitivity, specificity, and MCC, respectively. Similarly, Ge et al. [63] introduced a novel peptide information interpretation method known as chaos game representation which gives high dimensional feature vector while preserving bijection property. Such a technique works better in the case of identical sequence length which is the main drawback. To enhance the classification rate Akbar et al. [39] proposed cACP model based on Geary autocorrelation, conjoint traid, and Quasi-sequence alignment. Further, they deeply investigate different classifier algorithms for the prediction of ACPs and NCPs. As a result, they obtained 96.91%, 77.32%, 98.12%, 0.79 in accuracy, sensitivity, specificity, and MCC. Finally, we compared our empirical results with the latest work proposed by Ahmed et al. [64] that applies a convolutional neural network for the discrimination of ACPs and NACPs, but they show low accuracy because the deep learning model requires enough data for training. In this study, after comprehensive experiments, we proposed three models with a diverse number of *λ* values where the results verify that the proposed model with (*λ* = 1) achieved a high score as compared to the other existing approaches as shown in Table 6.

## 5. Conclusions and Future Research Direction

In this study, a trustworthy and intelligent framework for the proper categorization of ACPs is proposed. Four diverse feature extraction methods including AAC, DPC, TPC, and IPseAAC are integrated to obtain discriminative, representative, and robust information from peptides. Moreover, PCA is also used to remove homogenous characters from the resultant feature space. Finally, the proposed intelligent system is evaluated on two datasets using an ensemble classifier. After examining the projected outcomes of the classification learners, we discovered that our suggested model outperformed the current models in the literature utilizing both datasets. In the future, we aim to introduce a convolutional neural network and sequential learning models (long short-term memory (LSTM), gated recurrent unit, and bidirectional-LSTM) to further boost the performance and explore a larger dataset having multiple classes.

## Figures and Tables

**Table 1:** Existing approaches for the prediction of ACPs and NACPs using ML techniques.

References	Features	Evaluation	Classifier
[23]	PseACC, g-gap dipeptide	Accuracy, Sensitivity, Specificity, MCC	SVM
[24]	AAC, DPC, ATC, and PCP	------	SVM, RFT
[25]	g-gap dipeptide	Accuracy, Sensitivity, Specificity, MCC, F_1_-score	SVM
[26]	Composition-based, physicochemical properties and profiles	Accuracy, Sensitivity, Specificity, MCC, AUC, *p*-value	SVM, LR, KNN, RF
[27]	AAC, Conjoint triad, PAAC, GAAC	Accuracy, Sensitivity, Specificity, MCC, F_1_-score	SVM, RFT, LibD3C
[28]	K-space amino acid pair, Composite physiochemical properties, auto covariance,	Accuracy, Sensitivity, Specificity, MCC	SVM, RFT, FKNN
[29]	AAC, DPC, Terminus composition, binary profile	------	Tree based
[30]	Binary profile, DPC	Accuracy, Sensitivity, Specificity, MCC, AUC	SVM
[31]	ReduceAAC, AAC, average chemical shift	Sensitivity, Specificity, MCC, Q_A_	SVM
[32]	PAAC, RAAC, g-gap dipeptide	Accuracy, Sensitivity, Specificity, MCC	SVM, KNN, PNN, RF, GRNN
[33]	Pseudo position specific scoring matrix, Composite protein sequence, Split-AAC	Accuracy, Sensitivity, Specificity, MCC, G-mean, F-measure, Precision, Recall	SVM, KNN, PNN
[34]	Protein relatedness measure	Sensitivity, Specificity, MCC, AUC, Overall accuracy	SVM, AdaBoost
[35]	PAAC, Local alignment kernel	Accuracy, Sensitivity, Specificity, MCC	SVM

**Table 2:** The detailed statistics of ACP and NACPs of two peptide sequences datasets.

Datasets	ACP	NACPs	Total Data	Training Data	Testing Data
PSD_1_ (Benchmark) [23]	138	206	344	241	103
PSD_2_ (Main dataset) [39]	225	2250	2475	1732	743

**Table 3:** Empirical results over numerous collections of feature extraction techniques using PCA and ensemble classifier where bold value represents the best performance.

Method	PSD_1_				
	Accuracy	Sensitivity	Specificity	MCC	F_1_-Score
AAC	86.41	88.24	85.51	0.71	81.08
DPC	88.35	91.18	96.86	0.75	83.78
TPC	85.44	87.88	84.29	0.69	79.45
IPseAAC	88.35	83.33	91.80	0.75	85.37
AAC+DPC	92.23	92.11	92.31	0.83	89.74
AAC+TPC	90.29	84.09	94.92	0.80	88.10
AAC+IPseAAC	91.26	84.44	96.55	0.82	89.41
DPC+TPC	89.32	89.19	89.39	0.77	85.71
DPC+IPseAAC	93.20	88.37	96.67	0.86	91.57
TPC+IPseAAC	91.29	89.74	92.19	0.82	88.61
**Proposed Model (*λ* = 1)**	**97.09**	**97.44**	**96.88**	**0.94**	**96.20**
Proposed Model (*λ* = 2)	96.12	92.86	98.36	0.82	95.12
Proposed Model (*λ* = 3)	95.15	94.87	95.31	0.90	93.67

**Table 4:** Empirical results of numerous collections of feature extraction techniques over the main dataset using PCA and ensemble classifier where bold value represents the best performance.

Method	PSD_2_				
	Accuracy	Sensitivity	Specificity	MCC	F_1_-Score
AAC	88.96	58.82	93.76	0.53	59.41
DPC	91.25	68.42	94.60	0.61	66.67
TPC	86.27	48.94	91.68	0.39	47.42
IPseAAC	90.98	66.67	94.72	0.61	66.33
AAC+DPC	93.00	75.53	95.53	0.69	73.20
AAC+TPC	94.05	79.79	96.12	0.73	77.32
AAC+ IPseAAC	92.87	72.82	96.09	0.69	73.89
DPC+TPC	95.29	84.21	96.91	0.79	82.05
DPC+ IPseAAC	92.73	71.30	96.38	0.69	74.04
TPC+ IPseAAC	91.79	80.00	92.92	0.60	63.03
**Proposed Model (*λ* = 1)**	**98.25**	**96.77**	**98.46**	**0.92**	**93.26**
Proposed Model (*λ* = 2)	96.90	91.40	97.69	0.86	88.08
Proposed Model (*λ* = 3)	94.89	80.39	97.19	0.78	81.19

**Table 5:** Performance comparison of the proposed model with SOTA methods using PSD_1_ dataset, where the best result is highlighted in bold.

Model/Year	Accuracy	Sensitivity	Specificity	MCC	F_1_-Score
SPAP [62] 2013	87.00	92.00	86.00	0.74	-
LAK [35] 2014	92.68	89.70	85.18	0.78	-
iACP [23] 2016	95.06	89.86	98.54	0.89	-
IAP [31] 2016	93.61	89.86	96.12	0.86	-
iACP-GAEnsC [32] 2017	96.45	95.36	97.57	0.91	-
SAP [25] 2018	91.86	86.23	95.63	0.83	89.47
LDFM [27] 2020	92.73	87.70	96.10	0.84	92.70
**Proposed Model (*λ* = 1)**	**97.09**	**97.44**	**96.88**	**0.93**	**96.20**
Proposed Model (*λ* = 2)	96.12	92.86	98.36	0.91	95.12
Proposed Model (*λ* = 3)	95.15	94.87	95.31	0.89	93.67

**Table 6:** Performance comparison of the proposed model with SOTA methods using PSD1 dataset, where the best result is highlighted in bold.

Model/Year	Accuracy	Sensitivity	Specificity	MCC	F_1_-Score
NT5CT5 [30] 2013	92.65	74.67	94.44	0.61	-
GCGR [63] 2018	96.36	69.33	99.07	0.76	-
cACP [39] 2019	96.91	77.32	98.12	0.79	-
ACP-MHCNN [64] 2021	91.0	97.6	84.2	0.82	-
**Proposed (*λ* = 1)**	**98.25**	**96.77**	**98.46**	**0.92**	**93.26**
Proposed (*λ* = 2)	96.90	91.40	97.69	0.86	88.08
Proposed (λ = 3)	94.89	80.39	97.19	0.78	81.19

## Data Availability

Not applicable.

## Nomenclature

AAC	Amino acid composition	SVM	Support vector machine
ATC	Atomic composition	PCA	Principal component analysis
DPC	Dipeptide composition	ACPs	Anti-cancer peptides
MCC	Matthews correlation coefficient	NACPs	Non Anti-cancer peptides
ML	Machine learning	TPC	Tripeptide composition
RF	Random Forest	NB	Naïve Bayes
PseAAC	Pseudo amino acid composition	KNN	K-nearest neighbor
IPseAAC	Improved pseudo amino acid composition	LR	Logistic regressions
CD-HIT	Cluster database at high identity with tolerance	GRU	Gaited recurrent unit
LSTM	Long short-term memory	SOTA	State-of-the-art
